# A multidimensional phasor approach reveals LAURDAN photophysics in NIH-3T3 cell membranes

**DOI:** 10.1038/s41598-017-08564-z

**Published:** 2017-08-23

**Authors:** Leonel Malacrida, David M. Jameson, Enrico Gratton

**Affiliations:** 1 Laboratory for Fluorescence Dynamics, Biomedical Engineering Department, University of California at Irvine, Irvine, California USA; 2 Department Cell and Molecular Biology, University of Hawai’i at Manoa, John A. Burns School of Medicine, Honolulu, HI USA; 3 Área de Investigación Respiratoria, Departamento de Fisiopatología, Hospital de Clínicas, Facultad de Medicina, Universidad de la República, Montevideo, Uruguay; 4 Analytical Biochemistry and Proteomics Unit, Institut Pasteur of Montevideo, Montevideo, Uruguay

## Abstract

Mammalian cell membranes have different phospholipid composition and cholesterol content, displaying a profile of fluidity that depends on their intracellular location. Among the dyes used in membrane studies, LAURDAN has the advantage to be sensitive to the lipid composition as well as to membrane fluidity. The LAURDAN spectrum is sensitive to the lipid composition and dipolar relaxation arising from water penetration, but disentangling lipid composition from membrane fluidity can be obtained if time resolved spectra could be measured at each cell location. Here we describe a method in which spectral and lifetime information obtained in different measurements at the same plane in a cell are used in the phasor plot providing a solution to analyze multiple lifetime or spectral data through a common visualization approach. We exploit a property of phasor plots based on the reciprocal role of the phasor plot and the image. In the phasor analysis each pixel of the image is associated with a phasor and each phasor maps to pixels and features in the image. In this paper the lifetime and spectral fluorescence data are used simultaneously to determine the contribution of polarity and dipolar relaxations of LAURDAN in each pixel of an image.

## Introduction

With the advances in confocal and camera based microscopy in the last two decades, lifetime and spectral information are becoming common tools to study *in vivo* complex photophysical processes with high spatial and temporal resolution. The complementarity between these two observations, if measured together, could provide a deeper description of molecular interactions, metabolic profiles and membrane organization, among other properties^1, 2^. Analysis of these large and complex data sets from FLIM and hyperspectral measurements extensively use global analysis^3^ and deconvolution^4^. Currently, analyses of data from these complementary techniques are performed independently, which provide fragmented interpretation and could preclude a more basic description of the underlying photophysics.

The phasor plot approach is a model-free method to analyze and interpret lifetime and spectral resolved information from microscopy and cuvette experiments. Based on the mathematical derivation originally described by Weber at the early 1980s^5^, Jameson *et al*.^6^ introduced the phasor plot representation. Later Digman *et al*. further developed the phasor approach for fluorescence-lifetime imaging microscopy (FLIM) data^7^. More recently, Fereidouni *et al*. used the phasor approach for resolving fluorescence spectral components, specifically to unmix fluorescence signals from multiple dye staining and to interpret the data of FRET experiments without previous knowledge about the system under study^8–10^. Since these developments, phasor plots were applied in the biological field to analyze complex fluorescence processes^11–16^.

The combination of lifetime and spectral information in a single analysis (so-called spectral resolved FLIM, sFLIM) was previously proposed to analyze processes like FRET^17^, demixing multiple dye staining^18^ or for autofluorescence interpretation^19^. In these reports complex instrumentation and/or analysis were developed to obtain the combined information^18^. Examples of this approach are the spectral resolved fluorescence lifetime microscope with a multi-anode PMT, where seven^20^, sixteen^17^ or thirty-two^18^ parallel channels are used.

The phasor plot can provide a solution to employ simultaneously multiple lifetime or spectral data through a common visualization. Here we propose employing a specific property of phasor plots based on the reciprocal role of the phasor plot and the image. In the phasor analysis each pixel in the image has an associated point in the phasor plot and each point in the phasor plot maps to pixels and features in the image.

This simple approach that we call the Multidimensional phasor approach (MultiD-phasor), allow us to identify complex dynamic fluorescence events at pixel resolution, with simple mathematics and straightforward interpretation. Rather than using phasors for demixing spectral or lifetime components, in this work we are exploiting phasors to unravel complex dynamical processes occurring at cellular membranes due to photophysics affecting the spectral emission and the lifetime of the LAURDAN probe (LAURDAN: 6-Dodecanoyl-2-Dimethylaminonaphthalene). In particular we use spectral and lifetime information at each pixel of an image to detect and identify the polarity and/or occurrence of dipolar relaxation in membrane of NIH-3T3 live cells by the spectroscopic properties of LAURDAN^16^.

The dimethylaminonaphthalene family of probes was original designed to study the polarity and/or dipolar relaxation in macromolecular structures such as proteins and membranes^21^. The LAURDAN probe has several advantages compared with other dyes for membranes, among them the lack of segregation in membranes of different physical state^22^ and its exquisite sensitivity to the hydration and polarity at the membrane/water interphase^23^. For example, the spectrum of LAURDAN moves toward the green if the membrane is composed of shorter or unsaturated aliphatic chains or in the liquid disorder (Ld) phase of lipid bilayers but also dipolar relaxations move the spectrum toward the green. Therefore spectral information alone is insufficient to separate the effect of dipolar relaxations from changes of local polarity and the lengthening or shortening of the lifetime alone without spectral information cannot separate the shifting of the emission spectrum with time from the position of the spectrum due to different environments. In early cuvette studies, time resolved measurements of the entire emission spectrum was employed to separate different spectroscopic effect (polarity and dipolar relaxations)^24^. For images, the reconstruction of the time resolved spectrum at each pixel will require simultaneous acquisition of the spectral and the time decay and global analysis methods to identify the underlying photophysical phenomena. LAURDAN fluorescence can discriminate between polarity and hydration at the biological membranes interphase if properly analyzed^23^. Weber´s original report about dimethylaminonaphtalene probes in 1979, pointed out the potential of these dyes to discriminate independently the polarity and dipolar relaxation of their environments^21^. The first parameter is related to the apparent dielectric constant around the probe and the dipolar relaxation is related to the occurrence of molecular reorganization of dipole active molecules around the probe. In this sense, LAURDAN is located exactly behind the carbonyl group moieties of phospholipids, sensing the polarity and the hydration at this level^25^. From the point of view of the photophysics process, LAURDAN displays spectra and lifetime shifts related to its environment, making the analysis and interpretation sometimes difficult. Originally, LAURDAN spectral shift was analyzed in term of the generalized polarization function (GP)^22^, which measured the emission in two spectral bands and the analysis was done by normalized ratiometric measurement of the spectral shift. In the last decade, the lifetime and spectral shift for LAURDAN were analyzed using the phasor approach (lifetime and spectral phasor plots)^1, 16^. Phasor analysis of the lifetime and spectral components has the advantage of being a model-free approach and it uses simple algebra to combine components. The phasor plots allow us to discriminate between single or heterogeneous emission by visual inspection of the phasor plot without any previous knowledge or assumption. In the present work, we are proposing to expand the capabilities of the phasor approach to study the emission of LAURDAN in cells combining the spectral and lifetime information through a multidimensional phasor approach (MultiD phasor approach).

We are exploiting a mathematical property of the phasor plot that gives linear combination of 2 or more properties (lifetime components or spectral bands) if independent processes are occurring in a pixel. If we observe a linear combination of spectral features, in the same pixel there should be also a linear combination of lifetime components. The existence of a linear combination does not imply that the contribution of molecular species will be the same in the spectrum and lifetime plots, but still they should follow a linear combination since we are only assuming that if there are independent species giving rise to spectral components in one pixel, these species should also give rise to independent lifetime components.

**Figure 1:**
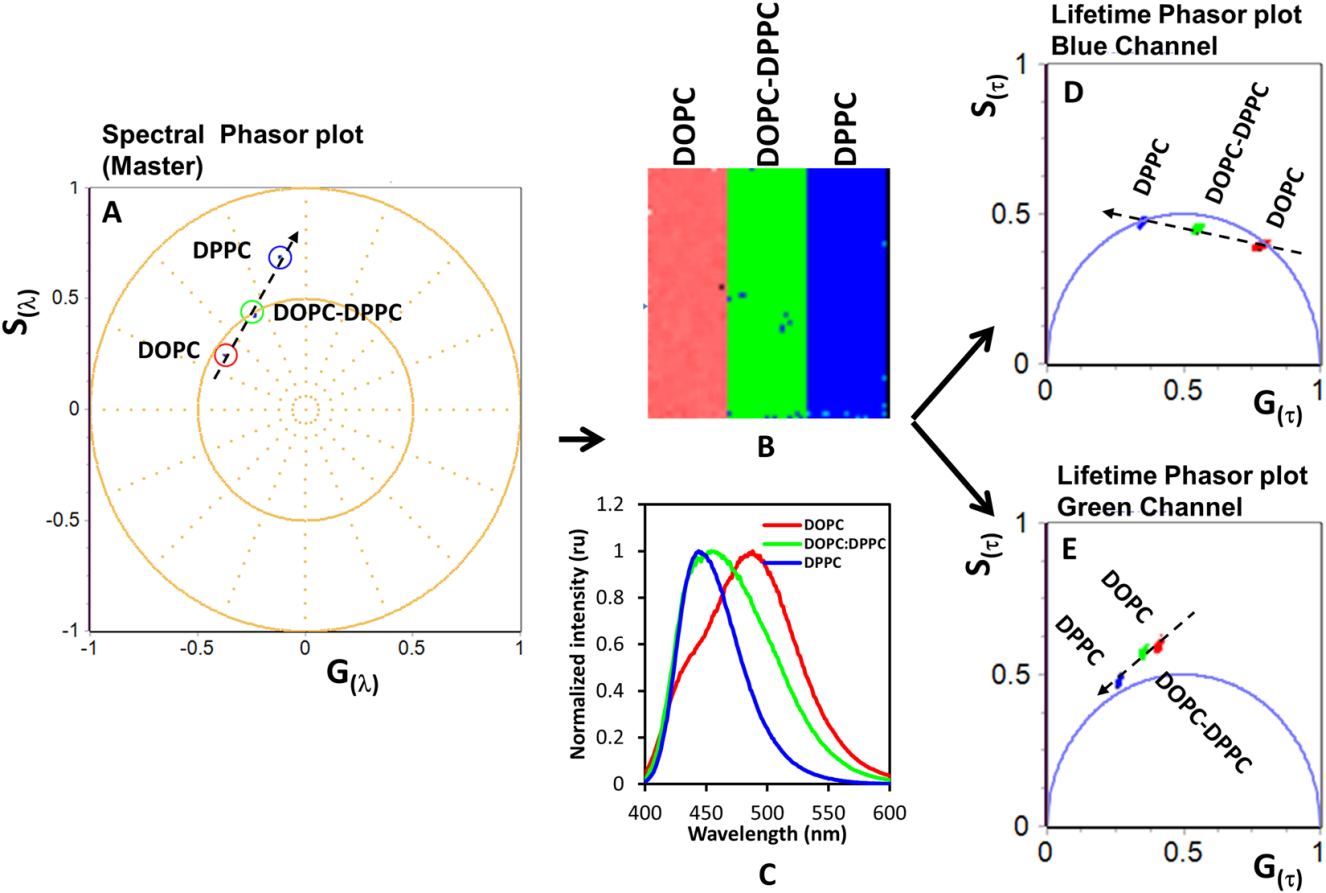
Schematic representation of the Multi-D phasor approach. Spectral and lifetime information were acquired in 3 samples in cuvettes. (**A**) After phasor transformation, the graph represents the spectral phasor. (**B**) The square in the center of the figure represents 3 pixels with different phospholipid composition. (**C**) Fluorescence spectra of LAURDAN in the 3 phospholipid mixtures used for this figure. (**D**,**E**) Graphs represent the phasors at two emission wavelengths. Selecting the spectral phasor plot as the master plot it is possible to identify in the common image (the 3 pixels at the center of the figure) the region selected by the 3 colored cursors in the master phasor plot. These pixels are highlighted using the same color code also in the lifetime phasor plots (**D**,**E**). A linear combination indicated by the dotted black lines in the master phasor plots gives linear combinations in the secondary lifetime phasor plots.

In the MultiD approach we use the spectra and lifetime information taken in the same focal plane by the connection between the image and the pixel position in the phasor plot (Fig. 1). In other words, if we plot the lifetime or spectral information in a phasor plot it is possible to come back to the original position in the image using a cursor selection. This approach can be done for as many dimensions as data are collected as shown in the Figure S1 in the supplementary material. In this paper we describe the basis of MultiD phasor approach and for the purpose of illustration we apply this method to a cellular system. It is beyond the purpose of this paper to show an extensive study of different cellular systems.

## Material and Methods

### Material

The (4-methyl-2-[4-(4-methyl-5-phenyl-1,3-oxazol-2-yl)phenyl]-5-phenyl-1,3-oxazole) (DM-POPOP) and all chemicals and solvents used were acquired from Sigma-Aldrich (St. Louis, MO-USA). 6-dodecanoyl-2-dimethylaminonaphthalene (LAURDAN) and medium for cell culture was acquired from Molecular Probes-Life Technologies (Thermo Fisher Scientific Inc. Huntington Beach, CA-USA). The 1,2-dipalmitoyl-phosphatidylcholine (DPPC), 1,2-dioleoyl-phosphatidylcholine (DOPC) and Cholesterol (Chol) were purchased from Avanti Polar Lipids Inc. (Alabaster, Alabama-USA).

### Cell Culture and treatments

Fibroblast derived from mouse (NIH/3T3 (ATCC® CRL-1658™)) were grown at 37 °C in 5% CO_2_ in Dulbecco’s modified Eagle’s medium (Thermo Fisher Scientific Inc. Huntington Beach, CA-USA) supplemented with 10% fetal bovine serum, 5 mL of Pen-Strep, and 2.5 mL of 1 M HEPES. Freshly split cells were plated onto 35-mm MatTek glass-bottom dishes (MatTek Corporation, MA-USA) coated with fibronectin 24 hours before the experiments. For the experiments of polarity/dipolar relaxation in membranes a final 1 μM solution of LAURDAN in the medium was used. Cells were incubated with LAURDAN for 30 min at 37 °C in the incubator and then the medium was replaced for a red phenol-free Dulbecco’s modified Eagle’s medium (Thermo Fisher Scientific Inc. Huntington Beach, CA-USA) before imaging.

### Steady state and lifetime fluorescence in cuvettes

Multilamellar vesicles (MLVs) were made with pure phospholipids (DOPC or DPPC) or with a mixture 1:1 of DOPC and DPPC. The final concentration of phospholipids was 0.5 mM. Lipid mixtures were prepared using a solution of Methanol-Chloroform (1:1 v/v) in a capped vial. Then solutions were transferred to conical glass vials and dried in a Savat SeepVac (Thermo Fisher Scientific Inc. Huntington Beach, CA-USA) for at least 1 hr at 40 °C to evaporate the organic solvent. After the lipids were dried, a dispersion of MLVs was prepared by adding a solution of a 5 mM buffer Tris with adjusted pH 7.0 and 150 mM NaCl at 45 °C. The samples were heated and vortexed at 50 °C for at least 1 h. The MLVs were labeled with LAURDAN in 1/500 molar ratio before drying; each sample was also prepared without probe as background solutions. The concentration of LAURDAN was determined using a molar extinction coefficient of 20,000 M^−1^cm^−1^ (at 364 nm in methanol).

LAURDAN spectra were measured on a Cary Eclipse Fluorimeter with a Peltier module (Agilent Technologies, Palo Alto, CA USA). The excitation used for LAURDAN was 360 nm and the emission spectrum was taken between 400 nm to 600 nm. Each experiment consisted of three independent samples plus the background mixture.

The multifrequency lifetime measurements were obtained using a Chronos FD Fluorometer (ISS, Champaign, IL USA). The modulation frequencies used were in the range 5–150 MHz. LAURDAN was excited with a 375-nm laser diode, using polarizers on the excitation and emission sides set to magic angles^26^. A bandpass filter F01-375/6–25 (Semrock, Rochester, NY, USA) was added in the excitation pathway. The blue and green components of the LAURDAN emission were viewed through Schott KV 399 + 404 bandpass or MK 500-longpass emission filters, respectively. To adjust the temperature, the cell holder was connected to a circulating water bath (Fisher Scientific, Pittsburgh, PA-USA). A temperature of 25 °C was used for the MLVs experiments. Dimethyl-POPOP dissolved in ethanol was used as a lifetime reference (1.45 ns) for the cuvette multi frequency measurements^27^.

### Lifetime and Spectral fluorescence microscopy

FLIM and spectral fluorescence data were acquired in a Zeiss LSM710 META Laser scanning microscope (Carl Zeiss, Dublin, CA-USA). The instrument was equipped with a Ti:Sapphire laser (Spectra-Physics Mai Tai, Newport Beach CA-USA) producing 80 fs pulses at a repetition of 80 MHz. An ISS A320 FastFLIM (ISS Champaign, IL) box was used to collect the lifetime decay data. A 40 × water immersion objective 1.2 N.A. (Carl Zeiss, Dublin, CA-USA) was used for all experiments. LAURDAN was excited using 780 nm wavelength. The emission was detected using two photomultiplier detectors (H7422P-40, Hamamatsu, Hamamatsu City, Japan) and a filter set to split the LAURDAN emission into two bands: a bandpass filter from Semrock (Rochester, NY-USA) was placed in front of each detector, 440/60 nm (Blue Channel) and 500/60 nm (Green Channel), and the signal was split using a 470 nm dichroic filter. For spectral images, the Lambda mode configuration of Zeiss LSM710 META Laser scanning microscope was configured in 32 channels, each with 9.7 nm bandwidth and a total range between 416 nm and 728 nm. Image acquisition in both FLIM and spectral mode was performed with a pixel frame size of 256 × 256 and the pixel dwell time used for FLIM or spectral images were 25.61μs/pixel or 5.09μs/pixel, respectively. FLIM and spectral data were processed using the MultiD-phasor routine in SimFCS software developed at the Laboratory for Fluorescence Dynamics (www.lfd.uci.edu). When indicated, denoising of the phasor plots was performed using the e-button algorithm of the SimFCS software. Calibration of the lifetime measurements was performed using a solution of 10 μM of Rhodamine 110 (Sigma-Aldrich Co., Milwaukee, WI-USA), using a reference lifetime of 4.0 ns (ISS webpage at www.iss.com).

### FLIM phasor plot analysis

For the lifetime phasor plot, the fluorescence decay I(t) was acquired at each pixel of an image and then following the rules of the phasor transformation^5^, the coordinates (*G*_(*τ*)_ and *S*_(*τ*)_) were calculated, (Equations 1 and 2). The phasor at each pixel was plotted in the polar plot (that we call Phasor plot) using the coordinates *G*_(*τ*)_ and *S*_(*τ*)_

1 $$x \mbox{-} \text{coordinate}={G}_{(\tau )}=\frac{{\int }_{0}^{T}I(t)\cos (\omega t){\rm{d}}t}{{\int }_{0}^{T}I(t){\rm{d}}t}$$

2 $$y \mbox{-} \text{coordinate}={S}_{(\tau )}=\frac{{\int }_{0}^{T}I(t)\sin (\omega t){\rm{d}}t}{{\int }_{0}^{T}I(t){\rm{d}}t}$$

where, *ω* is the angular modulation frequency, equal to 2π*f*, where *f* is the laser repetition frequency and *T* is the period of the laser frequency.

Phasors follow rules of vector addition and orthogonality, i.e., pixels that contain a linear combination of two independent fluorescent species will appear in the line joining the two independent emissions in the phasor plot.

### Spectral phasor plot analysis

The fluorescence spectra at each pixel was transformed in phasor coordinates (*G*_(*λ*)_) and (*S*_(*λ*)_) as described in equation 3 and 4, respectively^8, 14^. *I*(λ) represent the intensity at every wavelength (channel), *n* is the number of the harmonic and λ_i_ the initial wavelength. The, x and y coordinates were plotted in the spectral phasor plot.

3 $${\rm{x}}\,{\rm{c}}{\rm{o}}{\rm{o}}{\rm{r}}{\rm{d}}{\rm{i}}{\rm{n}}{\rm{a}}{\rm{t}}{\rm{e}}={G}_{(\lambda )}=\frac{{\int }_{{\lambda }_{min}}^{{\lambda }_{max}}I(\lambda )\,\cos \,(\frac{2\pi n(\lambda -{\lambda }_{i})}{{\lambda }_{max}-{\lambda }_{min}})d\lambda }{{\int }_{{\lambda }_{min}}^{{\lambda }_{max}}I(\lambda )d\lambda }$$

4 $${\rm{y}}\,{\rm{c}}{\rm{o}}{\rm{o}}{\rm{r}}{\rm{d}}{\rm{i}}{\rm{n}}{\rm{a}}{\rm{t}}{\rm{e}}={S}_{(\lambda )}=\frac{{\int }_{{\lambda }_{min}}^{{\lambda }_{max}}I(\lambda )\,\sin \,(\frac{2\pi n(\lambda -{\lambda }_{i})}{{\lambda }_{max}-{\lambda }_{min}})d\lambda }{{\int }_{{\lambda }_{min}}^{{\lambda }_{max}}I(\lambda )d\lambda }$$

The position for every pixel in the spectral phasor plot can be defined by the phase angle and the modulus (M) given the coordinates *G* and *S* (both for spectral or lifetime)

5 $$\phi =arctan({S}_{(\lambda )}/{G}_{(\lambda )})$$

6 $$M=\sqrt{{S}_{(\lambda )}^{2}+{G}_{(\lambda )}^{2}}.$$

The angular position in the spectral phasor plot relates to the center of mass of the emission spectrum and the modulus depends on the spectrum’s full width at the half maximum (FWHM). For instance, if the spectrum is broad its location should be close to the center. Otherwise, if there is a red shift in the spectrum, its location will move counterclockwise toward increasing angle from position (1, 0). Spectral phasors have the same vector properties as lifetime phasors. A detailed description of the spectral phasor plot properties can be found in Malacrida *et al*.^14, 16^.

### MultiD phasor plot analysis of LAURDAN fluorescence

The MultiD approach is based on a simple idea behind the connection between each point of the phasor plot and the pixels of the image. As previously discussed, it is possible to select a ROI in the spectral phasor plot and identify those pixels in the image selected in each ROI. If the area highlight at the lifetime image is common to the spectral data, then is possible to identify in the secondary phasor plot (lifetime phasor plot) where those pixels are located. The procedure behind the method is exemplified at the Figure S1. We acquire lifetime images in two channels and a spectral image at the same focal plane followed by the phasor transformation. Using the spectral phasor as the master plot, it is possible to obtain the location of the pixels selected by the cursor in the master plot in the secondary (lifetime) phasor plots.

## Results

### Lipid composition affects the position of a pixel in the phasor ploth

The procedure behind the MultiD method is explained in Fig. 1 where the data were taken with a spectrofluorimeter and a lifetime fluorometer in cuvette samples to exemplify a data set consisting of only a few pixels. We prepared 3 samples with different phospholipid composition, DOPC, DPPC and DOPC-DPPC (1:1 molar fraction) at 25 °C. Figure 1 show the schematics of the experiment where the square at the center of the figure represents 3 pixels of the image corresponding to the 3 cuvettes with the different phospholipid compositions.

From the spectral phasor plot for the different phospholipid mixtures (Fig. 1A) we deduce that these particular mixtures of pure phospholipids give a linear combination (black lines in Fig. 1A,D and E). In the spectral phasor plot, the DOPC sample has the redder emission (larger phase) and the DPPC sample the bluer emission (smaller phase angle). This figure shows that membranes of different lipid and their mixtures fall along a line in the spectral phasor plot (dashed line in Fig. 1A). In the two lifetime channels we also have a linear combination of the 3 mixtures (Fig. 1D and E, dashed lines). Namely, the lifetime in the blue channel becomes shorter due to increase in polarity induced by the addition of DOPC and the lifetime in the green channel becomes shorter and goes out of the universal circle due to the increase in dipolar relaxation allowed by DOPC. We note that only processes occurring during the excited state can bring the phasor outside of the universal circle, so that we can unequivocally determine that in this case there are dipolar relaxations. The spectral shift alone and the changes of the lifetime in the blue channel alone are not sufficient to establish the existence of dipolar relaxations, but the lifetime in the green channel is characteristic of dipolar relaxation in this case. This result shows that in every case we have linear combinations and that polarity and dipolar relaxations effects on the spectrum and lifetime values can be separated for each sample (or pixel in this case). Undoubtedly, the biological membrane is not as simple as the cuvette sample, but we should have linear combinations independently of the complexity of the system and we can make some general predictions about the effect of polarity and dipolar relaxations if we can compare spectral and lifetime behavior at each pixel in the biological samples as we did for this example using just 3 pixels.

Our analysis approach follows the following steps. First, in the spectral phasor plot we identify the pixels in the blue part of the spectrum, and then repeat for each part of the spectrum. Then for the pixels that have been identified to be for example in the green part of the spectrum, we look at their position in the phasor plot of the blue and of the green channel. If in the green channel the phasor is outside the universal circle, that result is interpreted as a membrane where dipolar relaxations are occurring. In other words, if we find the same or similar correlation in the cell than in the cuvette calibration we can recognize the effect of composition and phase order in the cell membrane for each pixel of the image.

### Independent phasor plot analysis of LAURDAN fluorescence in NIH-3T3 cells

**Figure 2:**
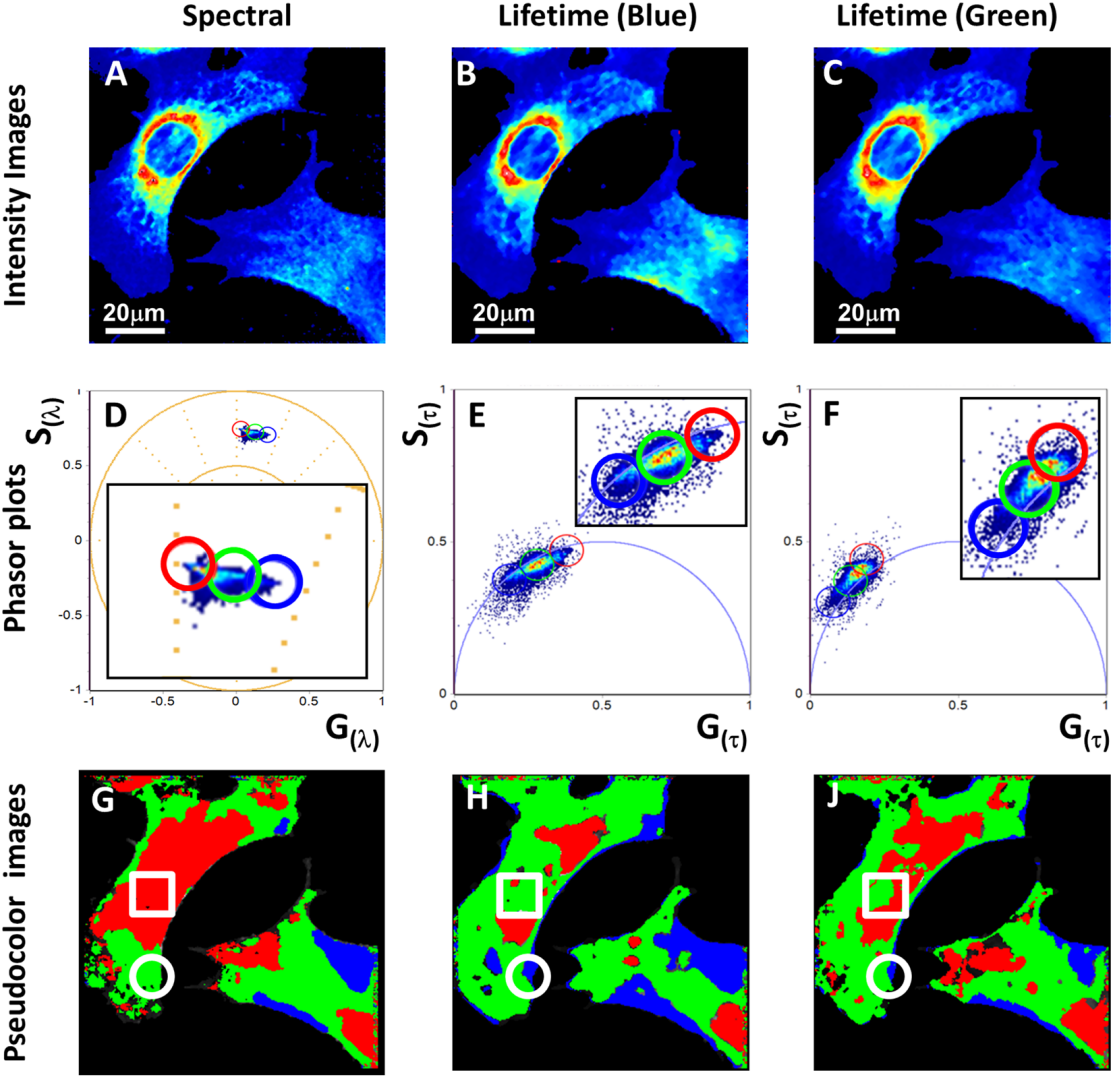
LAURDAN spectral and lifetime fluorescence information in NIH-3T3 cell membranes using independent analysis. (**A–C**) Intensity images of spectral (average of the 32 channels of the spectral detector) and lifetime (intensity of the blue and green channels filters, see Material and Methods for the channel transmission information), respectively. (**D–F**) Spectral and lifetimes phasor plots for the images in (**A–C**), insert at each phasor plot represent a zoom view of the LAURDAN trajectory in the phasor plots and cursor selection. A filter was applied to remove the noise only in the phasor plots. (**G,H,J**) images colored according to the color selection in the 3 independent phasor plots (**D,E,F**), respectively. Squares and circles show lack of coincidence in the color code by the independent cursor selection in the three phasor plots.

For the analysis of biological samples (Fig. 2) we acquired a 2-channel lifetime image (named blue and green channel, see Method section for the exact bandwidth) and emission spectra images (over 32 spectral bands) at the same focal plane in the cell. All data sets (FLIM in two channels and spectrum) were independently transformed in the phasor representation using equations 1–4 for each pixel of the image. Each pixel of the image (Fig. 2A,B and C) maps to a point in the 3 separate phasor plots (two lifetime phasors and one spectral phasor) but also each point in the phasor plots maps to a pixel (or several pixels) in the image (Fig. 2D,E and F). For example, for every phasor in the spectral phasor plot and the two lifetime phasor plots we identify one pixel in the image and the image is colored according to the color selected in the phasor plots. The coloring procedure is only used to visualize where a pixel with a given spectrum is located in the lifetime phasor plot. This simple approach gives the correspondence between the spectrum and the lifetime producing three color-code maps of the cell (Fig. 2G,H and I). However, following this procedure some pixels that are colored red in Fig. 2G have a different color in Fig. 2H and Fig. 2J. So using this approach, in which every image is treated independently, results in a mismatch of the spectral and lifetime colored maps. To assess if we can use the calibration lines in Fig. 1A,D and E to calibrate the pixels in the image, we need to determine if the same ordering of pixel (linear combinations) are also found in the cell image

As outlined in Fig. 1A, the spectral phasor plot displays a line of linear combination of lipid compositions and in the lifetime phasor plots we can easily identify the lines of linear combination where the same propriety is shown Fig. 1D and E (dashed lines). As anticipated in the case of a biological material, the same principle is not easily applicable to the cells. If we try to follow the principle used in Fig. 1 we can see that selecting points in the spectral phasor plot in the blue part of the spectrum (blue dots) we should have expect that these points will appear in the long lifetime region in the blue-channel lifetime plot (according to Fig. 1C, blue dots), instead we have a wide distribution in the blue-channel lifetime with all colors overlapping. One reason could be due to noise, but then denoising the data should bring the points on the proper line. Another reason could be that there is extra information in the lifetime phasor plot and that actually there is a linear combination but it is hidden by other effects that need to be recognized. In other words, there is sample heterogeneity that needs to be quantified and to extract this information it requires the analysis of both spectral and lifetime information in the same pixel. Of course, sample heterogeneity is not present in the cuvette data used for calibration.

For example, in the spectral phasor analysis, blue color highlights regions of blue emission (more solid) while red color indicates longer wavelength emission (more fluid). Analysis of spectral data alone can be interpreted in several ways. The blue painted regions in the images could indicate regions of low polarity or regions with low dipolar relaxation. Lifetime phasor plots could have an even more complex interpretation. For the blue channel, longer lifetimes are related to low polarity and for the green channel, apparent long lifetimes (as will appear measured by the time decay analysis) are associated with dipolar relaxation occurrences since the decay develops at later times (after excitation) in the green channel. Using the phasor plot, dipolar relaxations are unequivocally detected. Due to the delay after excitation in the green channel, the locations of the pixels in the phasor plot fall outside of the universal circle (see Fig. 2F), which can only happens for LAURDAN due to dipolar relaxations. From Fig. 2G it is possible to determine that the plasma membrane and more peripheral membranes show lower polarity and lesser dipolar relaxation than internal membranes indicating that heterogeneity could be at the origin of the discrepancy between the information in the spectral phasor and the lifetime phasor. A detailed inspection of the similarities and differences between spectral and lifetime channels allow us to determine that there are some correspondences with blue colored regions for the cell in the lower-right part of the image, but it is hard to get a general correlation between spectral and lifetime data by analyzing the 3 phasor plots independently.

### MultiD phasor plot analysis of LAURDAN fluorescence in NIH-3T3 cells

**Figure 3:**
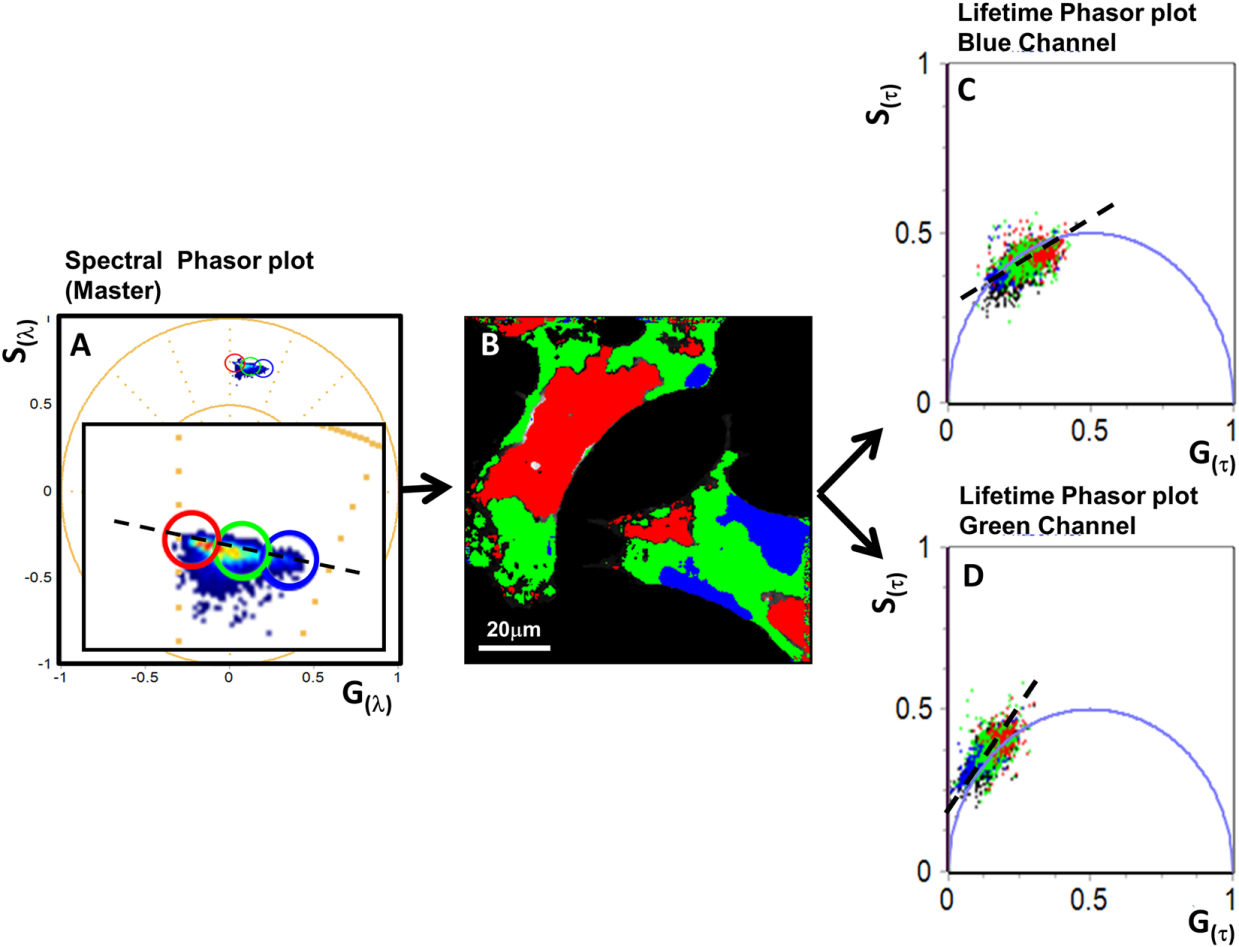
Multi-D phasor analysis of the LAURDAN fluorescence from NIH-3T3 cell membranes. (**A**) Spectral phasor plots (master) and cursor selection (red, green and blue) of different ROI in the phasor plot. The inner panel is the zoomed region of the phasor plot for better viewing. (**B**) Color-coded images generates by the cursor selection according to the master phasor plot. (**C**,**D**) Lifetime phasor plots (Blue and Green channels, see Material and Methods for the channel transmission information).

In Fig. 3 we show the basic idea of the MultiD analysis. Instead of using three independent phasor plots and 3 independent color maps we produce 3 phasor plots as in the previous section but we use only one image. Using the same image will allow us to display simultaneously several spectroscopic properties for the same pixel. For example, starting from the spectral phasor (defined as a master phasor plot, see Fig. 3A) we use the identical color code used in Fig. 2D but now we color code the phasors depending of the color they in the spectral color selection (Fig. 3C and D). An important consequence of the MultiD representation is that, for example, if we observe a linear combination in the master spectral phasor plot there should be an equivalent linear combination in each of the dependent phasor plots associated with a given image. Using the phasor approach and the color mapping of selected points in a different phasor plot we can identify the location in the image where the linear combination is found. These linear combinations could be hidden in one representation (which is the case for this image) but in the MultiD representation we can uncover all these linear combination by exploring the full range of measurements at once as shown in the next sections. Linear combinations arise when 2 or more independent molecular species are present in a pixel. If the molecular species are not interacting one with another then all the spectroscopic properties should reflect this linear combination although the fractional contribution to the linear combination could depend on the specific spectroscopic property.

In general, we can collect data at different excitations, emissions, polarizations, temperatures, etc., to build a MultiD phasor plot where we can correlate changes among the different data domains (lifetime, spectral and others). In supplemental Figure S1 we schematically illustrate this idea combining 3 lifetime data and 2 spectral data with different excitation wavelengths.

### Localization of lipid composition and fluidity in NIH-3T3 cells membranes with LAURDAN

#### First step in MultiD analysis: localization

In Fig. 3 we apply the MultiD analysis to the same data set used in Fig. 2. Using the selection of the corresponding pixel in the image (in the 3 phasor plots) afforded by the MultiD approach we can classify most of the pixels of the image. However, each pixel could contain the contribution of more species giving rise to linear combinations and these combinations should also be found in the other phasor plots establishing a better assignment of the properties of each pixel. After all, there should be only one consistent interpretation of the spectroscopic data but this aspect is missed in the independent analysis of the spectral and lifetime data done in Fig. 2. Using the spectral phasor as a master phasor plot (Fig. 3A), we highlight an ROI in the master phasor plot using a red cursor (longer wavelength of emission) and then we identify the location of the selected pixels in the original image (Fig. 3B) corresponding mostly to internal membranes. In the blue channel lifetime phasor plot, the pixels selected by the red cursor in the master spectral phasor (redder spectra) are a subpopulation of the shorter lifetime pixels. We can slice the entire spectral phasor distribution using different cursors as indicated in Fig. 3A. The blue cursor in the spectral phasor plot corresponds to phasors with longer lifetimes in the blue channel lifetime phasor plot (Fig. 3C). The pixels highlighted with each color in the images are not overlapping in the spectral phasor plot and they correspond to different LAURDAN environment at the membranes. But in the lifetime phasor plots the pixels are overlapping (Fig. 3C,D and supplementary Figure S2). For example, in Fig. 3C and D the green color is overlapping with the blue and red pixels. So pixels with the same spectrum (under the green cursor at the spectral phasor) don’t have the same lifetime (Fig. 3C and D, green pixels). In the green channel (Fig. 3D) all pixels are outside the universal circle characteristic of dipolar relaxations at those pixels.

### The importance of masking for detecting membranes with different LAURDAN properties

#### Second step in MultiD analysis: masking

**Figure 4:**
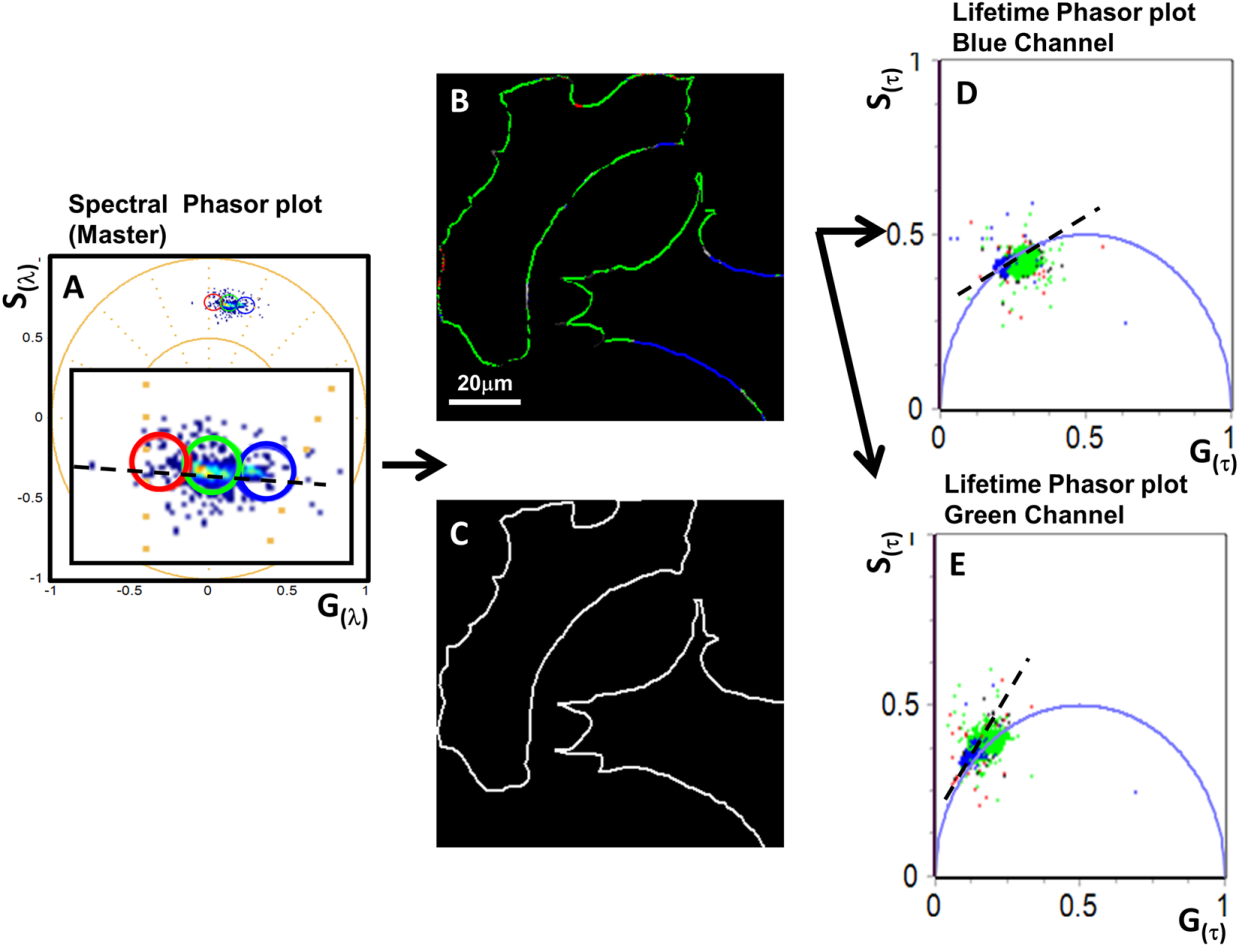
Multi-D phasor analysis of the LAURDAN fluorescence in NIH-3T3 plasma membranes. (**A**) Spectral phasor plots (master) and cursor selection (red, green and blue) of different ROI in the master (spectral) phasor plot. (**B**) Color-coded images generates by the cursor selection according to the master phasor plot. (**C**) Mask used by the analysis shown in **B**. (**D**,**E**) Lifetime phasor plots (blue and green channels, see Material and Methods for the channel transmission information).

Instead of analyzing all membranes of the cell together, subcellular analysis can be performed using masks from which a better understanding of the cell membrane organization is achieved (Fig. 4). Using the same mask (Fig. 4C) for the spectral and the lifetime channels, the masked image (Fig. 4B) shows that the plasma membrane has the lowest polarity and experience less dipolar relaxation than the rest of the cell (Fig. 4D and E). For the pixels selected by this mask, the lifetime phasor plots show that in the blue channel the pixels are almost on the universal circle and that for the green lifetime channel dipolar relaxation is present since the points are on or outside the universal circle. By analyzing regions of the cell using masks rather than the entire cell we can distinguish different behaviors that correlate with different membranes of the cell. In this case, the plasma membrane shows a relatively homogeneous population of relatively low polarity but with dipolar relaxations.

#### Third step in MultiD analysis: identification of processes in different membranes

**Figure 5:**
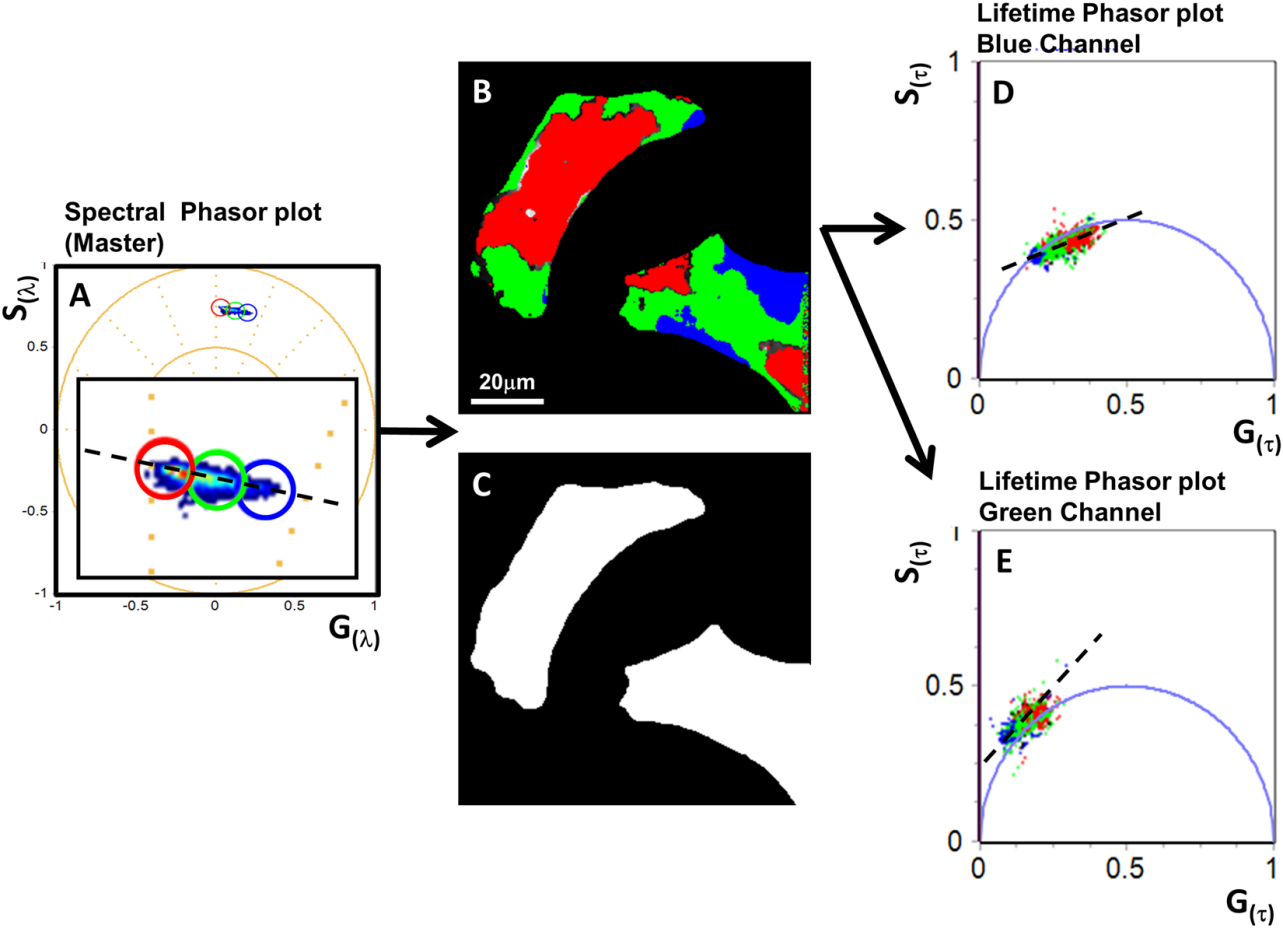
Multi-D phasor analysis of the LAURDAN fluorescence in NIH-3T3 internal membranes. (**A**) Spectral phasor plots (master) and cursor selection (red, green and blue) of different ROI in the phasor plot. (**B**) Color-coded images generated by the cursor selection according to the master (spectral) phasor plot. (**C**) Mask used by the analysis in (**B,D** and **E**) Lifetime phasor plots (blue and green channels, see Material and Methods for the channel transmission information).

In Fig. 5 we use a mask that selects all the pixels where internal membranes are located excluding the plasma membrane. Internal membranes show at least 3 spectral populations (selected by red, green and blue cursors (Fig. 5A and B), these pixels have different proportion of short to long lifetime in the phasor plot of the blue channel (Fig. 5D). The phasor plot of the green lifetime channel shows that the red cursor selects pixels with strong dipolar relaxations, while the blue cursor selects pixels with low polarity/dipolar relaxation. Since we don’t have an independent marker for each type of internal membrane we cannot unequivocally assign regions of the image to specific type of membranes and for the internal membranes we cannot assign specific LURDAN spectroscopic properties to each type of internal membrane but this assignment could eventually be attempted.

## Discussion

To illustrate the MultiD approach we stained NIH-3T3 with LAURDAN and acquired the lifetime and spectral data at the same confocal plane by 2-photon excitation. Figure 2A,B and C shows the intensity images of the spectral and lifetime (two channels) from LAURDAN fluorescence. As previous reported by our group^2, 16^ in a cellular system, LAURDAN show a distribution in the spectral phasor explained by the fact that LAURDAN molecules are in different membrane environments (Fig. 2D), presumably fluid and ordered membranes. Between the extreme situation of pure fluid and ordered membrane, it is possible to observe pixels in the line joining the two extreme membrane environments. These pixels reflect linear combinations between different proportions of fluid and order membranes rather than the existence of a different membrane phase. **Step 1: localization**. Using a color-code cursor selection in the spectral phasor plot, it is possible to identify the locations of membranes within the range fluid-order (Fig. 2D). The NIH-3T3 cells show that a bluer spectrum is associated with more peripheral membranes. It is thought that this peripheral location reflects cholesterol distribution which allows the occurrence of high packing membrane assemblies, or liquid order organization at the plasma membrane. On the other hand, the interior of the cells present a high degree of different linear combination of fluid/order membranes. Previously it was proposed that the independent phasor analysis of the blue and green lifetime emission of LAURDAN, can allow to discriminate between polarity and dipolar relaxation occurrence in the LAURDAN moieties^1^. In Fig. 2E and F it is possible to see the pixel distribution in the phasor plot for LAURDAN emission in the blue and green channels (Fig. 2E and F, channels-1 and -2 respectively). The blue channel phasor plot shows the distribution between a single exponential decay (defined by its position on top of the universal circle), which reflects the change in terms of polarity between pure fluid and order membrane. Additionally, pixels in the green lifetime channel are outside the universal circle, because of the occurrence of a delay in the fluorescence emission explained by the water dipolar relaxation around the LAURDAN probe^16^. Although it is possible to see correlation between the blue-green pixels location in the spectral image with the corresponding location in the blue and green lifetime channels, it is also clear that there are differences. This observation pointed out that the independent analysis is missing the connection at the pixel level given by the spectral property and the lifetime values for the same pixel.

In Fig. 3, the same data was analyzed by the MultiD-phasor approach. Defining the spectral phasor plot as a master phasor plot, it is possible to select regions in the phasor plot and highlight the cell region that contains these particular LAURDAN spectra (for example blue spectra, Fig. 3). At the same time, it is possible to look at the secondary lifetime phasor plots where the same pixels selected in the spectral phasor plot are located. Panels C and D in supplementary Figure S2 shows the correlation between the shifts indicated by the cursors red, green and blue, and their correspondence with the spectral shift from red to blue and short to long lifetime (respectively). Interestingly, it is possible to identify how the spectral phasor plot (red and blue cursor) selects those pixels in the blue channel that are on top of the universal circle. These LAURDAN environments reflect membranes with high (red) to low (blue) polarities, and the green cursor highlight those pixels that have different proportions of linear combination of low and high polarities (see Fig. 3 and Figure S2 in the supplementary material). The location of the relatively low polarity LAURDAN environments (green cursor) is associated almost exclusively with the plasma membrane; instead the high polarity environments are all located in the interior of the cell. The relationship between the spectral phasor and the green lifetime channel follows a similar profile as the blue channel, with the difference that for the green lifetime channel all the pixels are located outside the universal circle (Fig. 3D). Even the low polarity membranes which have long lifetimes in the blue channel are located outside of the universal circle in the green lifetime channel, underlying the occurrence of dipolar relaxation also in the more ordered membranes. However, from the position of the pixels associated with a low polarity in the blue lifetime channel (that also have blue spectra) we can conclude that at these pixels there is less dipolar relaxation than in the ones selected by the red cursor in the spectral phasor and high polarity in the lifetime blue channel.

**Step2: masking**. In order to better determine the locations at which different LAURDAN’s photophysics occurred we used masks to analyze the data in the different membranes of the cell. Masking was used to separate peripheral cell membranes from internal membranes. Figures 4–5 show the masking process done to discriminate between the membranes. The color-coded images show unequivocally the correlation between outer membranes and the spectrum color (Figs 4 and 5). From the color code used along the LAURDAN trajectories we can see the locations of these environments in the cell (Fig. 4). The same analysis can be done using the blue and green lifetime channels as we show in Fig. 4. Plasma membrane is associated with the low polarity membrane and with lower dipolar relaxation.

**Step 3: processes**. By the correlation achieved with the MultiD approach, it is possible to identify separately the contribution to polarity from the dipolar relaxation at the same pixel. Taking advantage of this approach in Figs 4 and 5, we determined where exactly the pixels with low polarity/high polarity in the blue lifetime channel phasor plot are located. LAURDAN trajectory in the green lifetime phasor plot is outside the universal circle. This change in the phase is driven by the occurrence of dipolar relaxations.

## Conclusions

We describe the MultiD analysis pipeline that allows us to use different phasor plots obtained in different domains (spectral and lifetime at 2 different emission bandwidth) to determine the fluorescence properties of LAURDAN at each pixel of a image. In few words, the purpose of the MultiD analysis is to find if correlations seen in one phasor plot (the master plot) are also found in the phasor plots of other fluorescence properties. This correlation is generally seen as a linear combination of spectral (or lifetime properties) in the master phasor plot. Only few pixels in an image could show the correlation and we propose a method based on masks to isolate these pixels form the rest of the pixels in the image. In our specific example, if we were to use only the spectral properties we would not be able to separate the effect on LAURDAN fluorescence due to different compositions of the membrane from the effect due to changes in membrane water penetration that result in the phenomenon of dipolar relaxations. A characteristic signature of dipolar relaxation is the position of the lifetime phasor on the green channel outside the universal circle. The proposed MultiD-phasor approach allows us to identify the connection between spectral and lifetime changes unequivocally based on the linked connection between both processes as shown in the microscope image in the locations where dipolar relaxations are found. If we analyze all pixel of cell image together we cannot identify the linear combinations that we observe in model systems. By masking several distinct regions of the cell, in the example of this work we show regions in the plasma membrane where there are different lipid compositions and regions where there are strong dipolar relaxations, providing a specific spectroscopic signature for each region of a membrane. Although in this paper we only used 2 masks since we did not have a marker for different membranes, we could find spectral signatures unique to each cell membrane.

We can think of the MultiD phasor analysis as a form of global analysis for different spectroscopic properties applied to images. However, while globals analysis methods ultimately use a mathematical model to fit the data, the MultiD phasor approach is a fit-free method that exploit correlation between different phasor plots, where the correlations are due to linear combination of properties in the same pixel and the same linear combination is recognized in the different phasor representations, in this work, the lifetime phasors. We could have used a 3-dimensional representation of spectral and lifetime phasor, for example using a vertical axis to represent the lifetime phasor and the horizontal plane for the spectral phase, but we would run out of dimensions as we add more fluorescence properties. Instead the MultiD method described in this work can be applied to an arbitrary number of dimensions (independent measurements). The basic idea is that if a linear combination exists in one dimension, the same linear combination could exists (or not) among other fluorescence properties. In this paper we indicate a pipeline of how to propagate the linear combination from one phasor plot (the master) to the other phasor plots. Using this principle we were able to identify pixels in the plasma membrane of a live cell where dipolar relaxations occur and correlate these pixels with the lipid local composition. Although the method proposed has pixel resolution, in practice S/N considerations require that several adjacent pixels must have similar properties otherwise it will be difficult to distinguish with specific fluorescence signatures.

## Electronic supplementary material

Supplementary Information
