# Supplementary material for: A multidimensional phasor approach reveals LAURDAN photophysics in NIH-3T3 cell membranes
Source: Sci Rep. 2017 Aug 23;7:9215. doi: 10.1038/s41598-017-08564-z (PMC5569084; doi:10.1038/s41598-017-08564-z)
Supplement: Supplementary file 1 — Supplementary Information [file 41598_2017_8564_MOESM1_ESM.pdf]

Supplemental material

A multidimensional phasor approach reveals LAURDAN photophysics in NIH-3T3 cell membranes.

Leonel Malacrida, David M. Jameson and Enrico Gratton

Supplementary Material

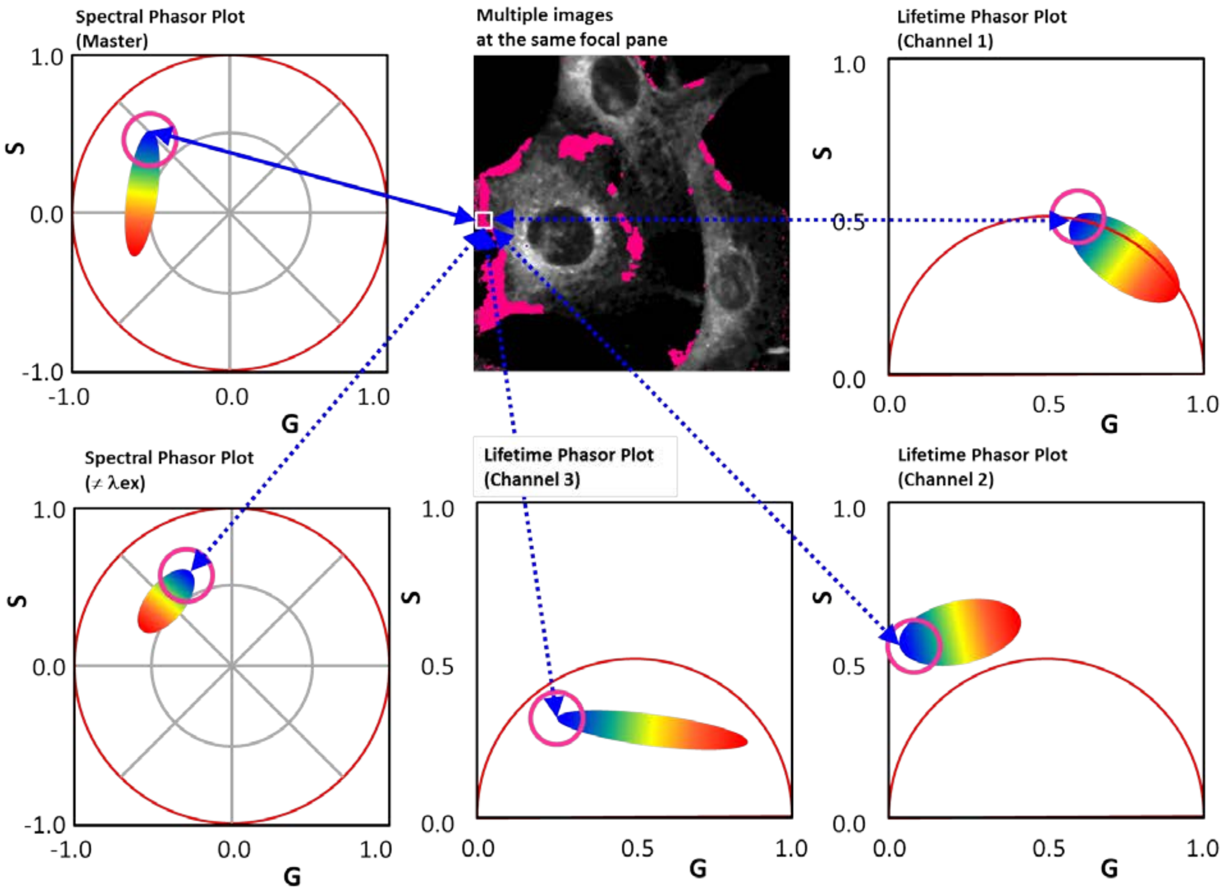

Figure S1: Scheme of the Multi-D phasor approach. The cartoon represents the idea behind the multidimensional connection through the image between n-dimension of fluorescence's, in this case 5 dimensions. In this schematic illustration we start with the emission spectral image that is connected to other emission spectra obtained at different excitation (≠ λex) and to 3 lifetime channels obtained using 3 different filters. Many lifetime channels and spectral fluorescence and/or different excitation/emission wavelengths can be used in the multi-D approach. The arrows represent the phasor transformation and allow connecting the master plot to the other plots at the same pixel.

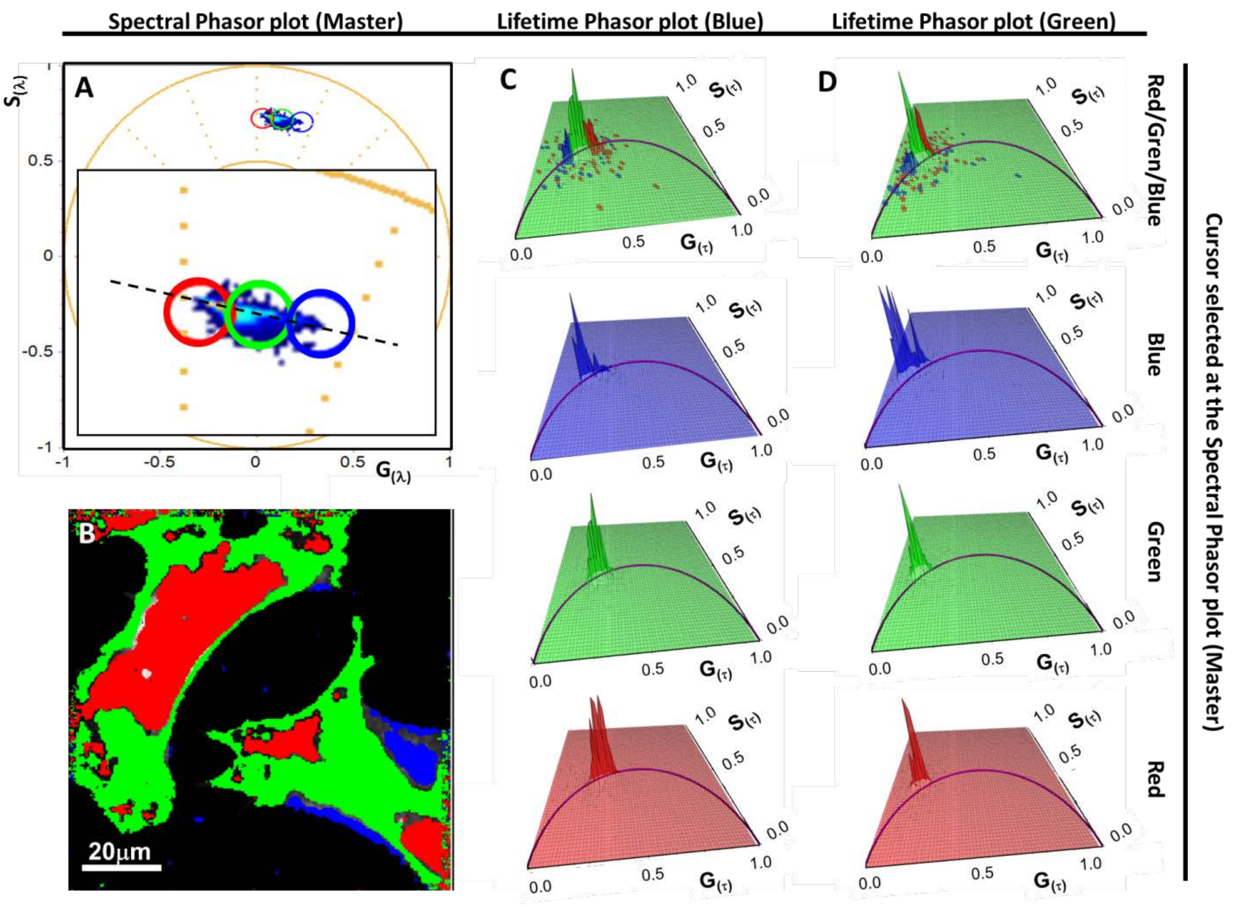

Figure S2: Multi-D phasor analysis of the LAURDAN fluorescence from NIH-3T3 cell membranes. A) Spectral phasor plots (master) and cursor selection (red, green and blue) of different ROIs in the spectral phasor plot. B) Color-coded image generated by the cursor selection of the master phasor plot (Spectral, A). C and D) 3D lifetime phasor plots (blue and green channels (see Material and Methods for the channel transmission information). From top to bottom the phasor plots are organized by the respective cursor selection in the master phasor plot (all cursors, blue, green and red, respectively). The pixels in the secondary phasor plots are colored using the same color code selected by the cursor at the master phasor plot.
